# Perioperative management of a patient with tuberculous destroyed lung and ascending aortic aneurysm undergoing a Bentall procedure: a case report

**DOI:** 10.3389/fcvm.2026.1762499

**Published:** 2026-02-19

**Authors:** Huimin Liu, Qing Li, Sijia Wang, Weidong Fu, Zhujun Huang, Mingzhi Zheng, Liu Luo, Lin Tang

**Affiliations:** 1 Department of Anesthesiology, Zhuzhou Hospital Affiliated to Xiangya School of Medicine, Central South University, Zhuzhou, Hunan, China; 2 Department of Anesthesiology, Zhuzhou Clinical College, Jishou University, Zhuzhou, Hunan, China

**Keywords:** ascending thoracic aortic aneurysm (ATAA), Bentall procedure, cardiopulmonary bypass (CPB), multimorbidity, tuberculosis destructive lung (TDL)

## Abstract

**Background:**

The coexistence of tuberculous destroyed lung (TDL) and ascending aortic aneurysm represents a rare multimorbidity, posing significant challenges for perioperative management in patients requiring cardiopulmonary bypass (CPB)-assisted major vascular surgery. The clinical manifestations of this polycoexisting disease may be asymptomatic or present as symptoms of one of the diseases or both. Currently, there is no universally accepted diagnostic protocol available for the definitive diagnosis of complex conditions such as tuberculosis and its comorbidities.

**Case presentation:**

A 58-year-old woman presented with a 1-month history of cough, expectoration, chest tightness, and dyspnea, which had exacerbated over the preceding week. Pulmonary computed tomography revealed left lung fibrosis with architectural destruction, along with aneurysmal dilation of the ascending aorta. Her medical history included tuberculosis (diagnosed 20 years earlier and treated with anti-tuberculosis therapy for 6 months), hypertension (managed with levamlodipine besylate 5 mg daily), and recurrent pulmonary infections. After multidisciplinary evaluation, she underwent an elective Bentall procedure under CPB. Postoperatively, there was no evidence of tuberculosis reactivation or major complications, and she was discharged after 23 days of hospitalization.

**Conclusions:**

Successful surgical intervention for TDL combined with thoracic aortic aneurysm is rarely reported. Early diagnosis and timely surgery are critical for improving patient outcomes. This case highlights the importance of multidisciplinary collaboration and customized perioperative strategies in managing such complex multimorbidities.

## Background

Tuberculous destroyed lung (TDL), a severe sequela of pulmonary tuberculosis, is characterized by irreversible parenchymal destruction, fibrosis, and cavitation, leading to significant functional impairment (1). Concomitant thoracic aortic aneurysms (TAAs), particularly those involving the aortic root with aortic valve insufficiency, present life-threatening risks due to potential dissection or rupture (2). Given the aortic valve insufficiency resulting from the aneurysm affecting the valve and the multiple calcifications observed during surgery, composite graft replacement—the Bentall procedure, which is the gold standard for treating such conditions—was selected as the surgical approach (3). In China, where both tuberculosis and cardiovascular diseases are prevalent, the coexistence of these conditions demands innovative perioperative approaches. This report details a unique case of thoracoabdominal aortic dissection (TDL) complicated by an ascending aortic aneurysm, which was effectively managed through the implementation of the Bentall procedure.

## Case presentation

A 58-year-old female patient presented with symptoms of cough, expectoration, chest tightness, and shortness of breath that persisted for over a month, which had worsened during the week prior to her hospital visit. She was admitted to the Department of Respiratory Medicine at our hospital on April 21, 2024. The patient had been diagnosed with tuberculosis 20 years earlier, for which she had received a year of regular anti-tuberculosis treatment before discontinuation. Since then, the patient has not undergone regular treatment. She also had a 10-year history of hypertension, for which she and was taking levamlodipine besylate 5 mg once daily. One month prior to admission, she developed cough without any obvious cause with a small amount of white foamy phlegm, accompanied by chest tightness and shortness of breath. There were no symptoms such as chills, fever, headache, dizziness, nausea, or vomiting. She self-medicated with amoxicillin and levofloxacin tablets for infection treatment, and consumed salvia miltiorrhiza tablets for cardiac protection treatment. Her symptoms were alleviated, but recurred sporadically. The patient reported no family history of related diseases.

**Figure 1:**
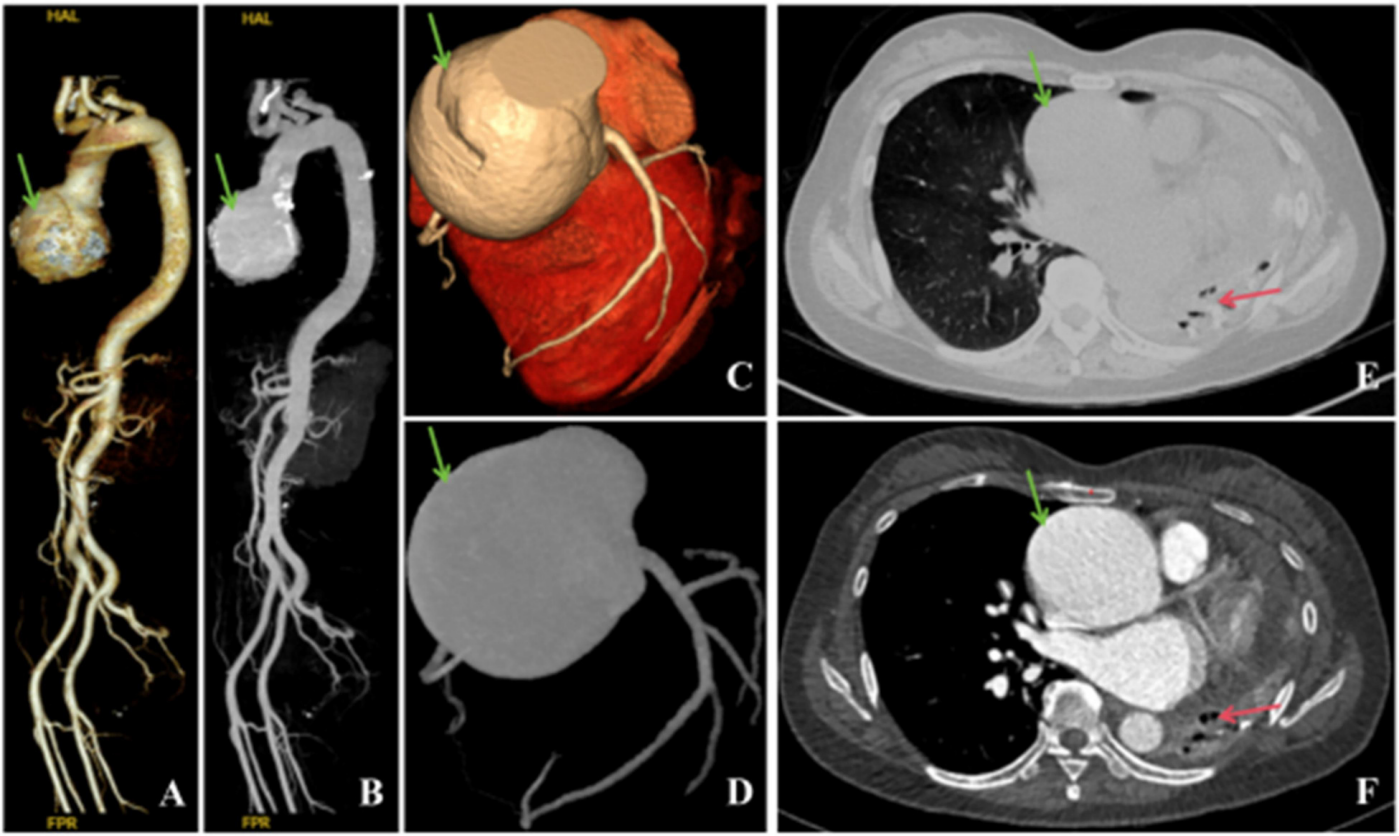
Preoperative thoracoabdominal aorta and coronary artery CTA, thoracic enhanced CT. **(A,B)** Ascending aortic aneurysmal dilation, tortuous course of the thoracic aorta (indicated by the green arrow), no abnormality observed in the abdominal aorta. **(C,D)** Coronary CTA: Ascending aortic aneurysmal dilation (indicated by the green arrow), myocardial bridge in the distal segment of the left anterior descending artery. **(E,F)** Aneurysmal dilation (indicated by the green arrow) with left lung destruction (indicated by the red arrow), secondary pulmonary tuberculosis in the right lung, a small amount of effusion in the left pleural cavity, and cardiac enlargement.

For further treatment, the patient visited the outpatient department of our hospital and underwent a chest enhanced computed tomography (CT) scan after admission (Figures 1E,F). The scan revealed left lung destruction, secondary pulmonary tuberculosis in the right lung, with lesions mainly characterized by fibrosis, proliferation, and calcified changes, a small amount of effusion in the left pleural cavity, an enlarged heart volume, and a small amount of effusion in the pericardial cavity. At the onset of the illness, the patient's symptoms of coughing and chest tightness with shortness of breath worsened, accompanied by a decrease in appetite.

The patient’s admission physical examination revealed vital signs within normal ranges for an adult, with a body temperature of 36.9 ℃, heart rate of 70 beats per minute, respiratory rate of 19 per minute, blood pressure of 143/57 mmHg, and SpO_2_ 97% without supplemental oxygen. The patient appeared to suffer from a chronic illness, but without edema in the eyelids and facial area, enlarged superficial lymph nodes palpable, or deformity of the thorax. Auscultation of the left lung did not reveal any breath sounds, while the right lung had slightly diminished breath sounds without any dry or wet rales. A high-pitched, sighing decrescendo murmur was heard in the aortic valve area, consistent with the characteristics of aortic stenosis, where the murmur typically begins with the onset of systole, increases in intensity to a mid-systole peak, and then gradually diminishes toward the end of systole. Abdominal examination showed no gastrointestinal shape or peristaltic waves, no tenderness or muscle tension in the entire abdomen, no palpable masses, and negative shifting dullness. The laboratory examination showed the following: platelets 403 × 109/L, pro-BNP 5,267 pg/mL, TnI 0.046 µg/L, ESR 24 mm/h, UA 383 μmol/L, and mycoplasma pneumoniae total antibody (1:40) positive. The results of sputum tuberculosis culture, PCR, T-SPOT, and tuberculosis DNA were negative. Urine occult blood was positive (2+). Arterial blood gas analysis revealed a pH of 7.43, PCO_2_ of 37 mmHg, and PaO_2_ of 78 mmHg. ECG demonstrated sinus tachycardia with left ventricular hypertrophy.

Coronary CTA revealed ascending aortic aneurysmal dilatation and left anterior descending distal myocardial bridge (Figures 1C,D). Echocardiography revealed enlargement of the left ventricle, which can be attributed to several potential causes, such as hypertension, coronary heart disease, valvular heart disease, cardiomyopathy, and congenital heart defects. Dilation of both the left ventricle and right atrium, aneurysmal dilation of the ascending aortic root, severe aortic regurgitation with pericardial effusion. Bronchoscopy was not performed due to the patient's refusal. Thoracoabdominal aortic CTA showed ascending aortic aneurysmal dilatation, tortuous thoracic aorta, and no significant abnormalities in the abdominal aorta (Figures 1A,B). The echocardiogram showed aneurysmal dilation of the aortic sinuses and ascending aorta, left ventricular enlargement (left atrium 47 mm, left ventricle 57 mm), severe aortic valve regurgitation, mild regurgitation of the mitral/tricuspid valves, normal range of left ventricular systolic function, reduced diastolic function, and a small amount of pericardial effusion. The systolic pulmonary artery pressure was approximately 50 mmHg.

Following comprehensive multidisciplinary consultation and analysis of the patient's symptoms—including persistent cough, sputum production, chest tightness, and shortness of breath—along with postadmission diagnostic tests, the final diagnosis was established as aortic root aneurysmal dilation, severe aortic valve insufficiency, TDL, and mycoplasma infection. In view of the patient's significant aortic dilation (diameter about 72 mm), there was a risk of aortic dissection and rupture, which were considered life-threatening. Recommendation was provided to limit activity along with clarification of surgical indications. The patient tested negative for sputum tuberculosis culture, PCR, T-SPOT, and tuberculosis DNA, with no evidence of active pulmonary tuberculosis. Because of the patient's inability to cooperate, pulmonary function tests were not performed. Once the patient's pulmonary mycoplasma infection improved and preoperative preparations were completed, elective Bentall surgery was performed under general anesthesia.

**Figure 2:**
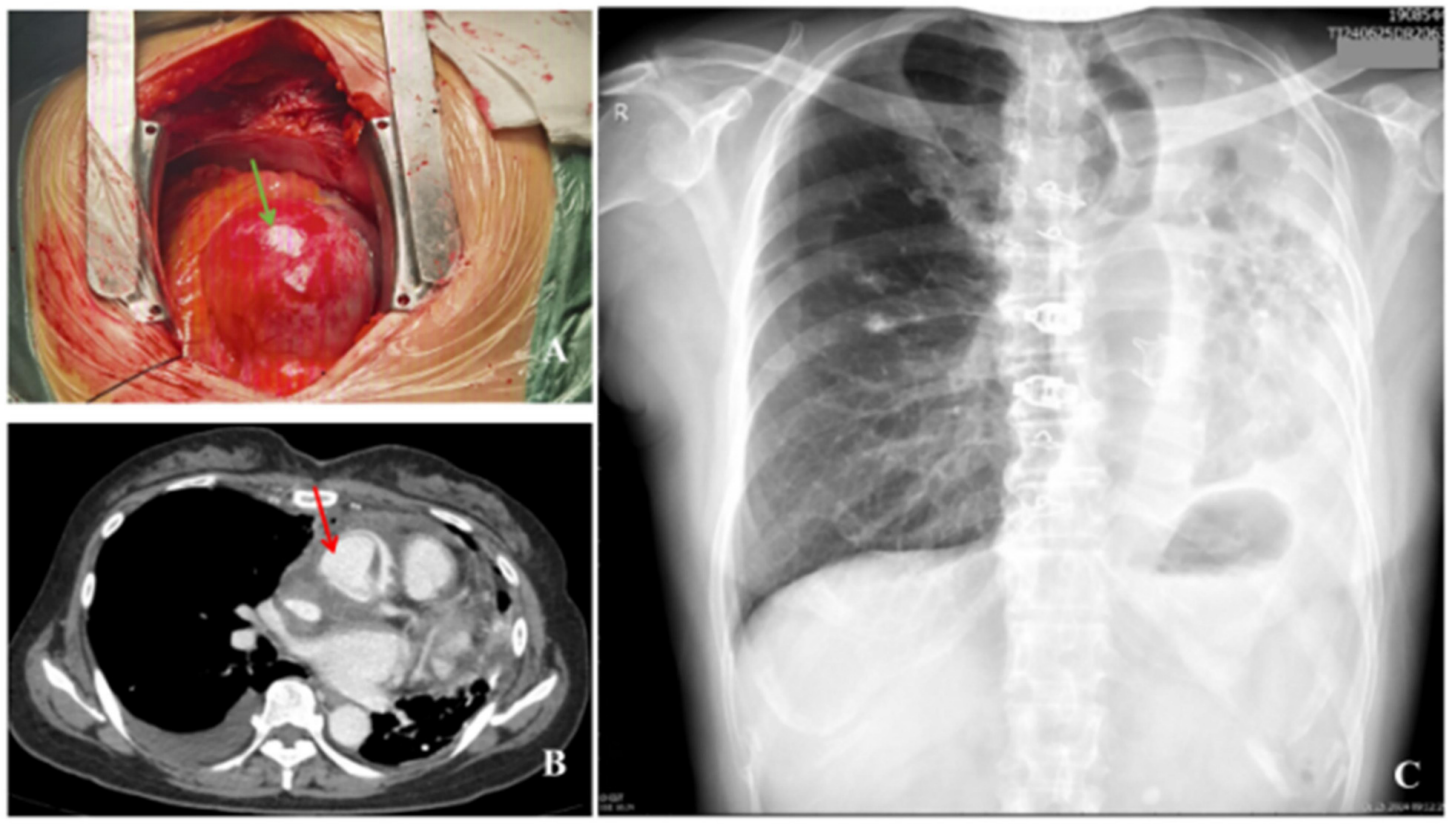
Intraoperative findings and postoperative cardiovascular CTA and chest x-ray images. **(A)** The aneurysm observed during the operation (indicated by the green arrow). **(B)** Postoperative changes of the ascending aorta graft seen on chest CTA (indicated by the red arrow), with no significant changes in the left lung destruction. **(C)** Chest X-ray 1 month after surgery shows left lung destruction, compensatory emphysema in the right lung, and postoperative changes.

Prior to surgery, her vital signs were stable: heart rate (HR) 83 beats per minute, BP 132/68 mmHg, RR18 breaths per minute, SpO_2_ 96% (without oxygen), and ECG demonstrating sinus rhythm with regularity. Upon entering the operating room, the patient was immediately connected to an ECG monitor, and intravenous access was established. Mask oxygen therapy (oxygen concentration of 100%, 6 L/min) was provided, increasing SpO_2_ to 98%. After induction of anesthesia, the patient was placed in the supine position. Routine disinfection and draping were performed. The right femoral artery was isolated and prepared for use. A median sternotomy incision was then performed. The ascending aortic root exhibited aneurysmal dilation, with a diameter measuring approximately 72 mm (Figure 2A), exceeding the normal range for this segment of the aorta. No ulcer or damage was visible on the blood vessel wall. The aortic annulus was enlarged, and the aortic valve leaflets were thin with multiple punctate calcifications. A large amount of light yellow pericardial effusion was also noted, along with significant enlargement of the left atrium and ventricle. Subsequently, an inverted “T” pericardiotomy was performed, and 22,000 units of heparin were administered intravenously. Femoral artery and cavoatrial cannulation were established for extracorporeal circulation. Cardiopulmonary bypass (CPB) was initiated after the activated clotting time (ACT) exceeded the normal range of 480–1,200 s, with cooling to 30 ℃. A left ventricular vent was placed via the right superior pulmonary vein. Ventilation was discontinued, and the aorta was clamped approximately 5 cm distal to the tumor. Subsequently, the aorta was incised, and a solution of histidine-tryptophan-ketoglutarate was injected under direct vision to induce cardiac arrest. Ice was placed in the pericardial cavity, and the diseased aortic valve and aneurysm were excised. A valve conduit constructed with a bioprosthetic valve (Medtronic) and an artificial vascular graft (Medtronic) was intermittently sutured at the aortic valve site. The native coronary artery was transplanted to the coronary artery orifice and continuously anastomozed with the artificial graft using 5-0 polypropylene sutures, following the standard Button Bentall procedure. After reconstruction of the aortic root, a perfusion test was performed, demonstrating good perfusion of the aortic root coronary artery without leakage. The distal end of the artificial graft was continuously anastomozed with the distal end of the ascending aortic aneurysm using 4-0 polypropylene sutures to reconstruct the artificial ascending aortic conduit.

After repayment of oxygen debt, air evacuation, and aortic unclamping, rewarming was initiated to raise the body temperature above 35 ℃. The heart resumed sinus rhythm after electrical defibrillation. In cases of suspected coronary flow compromise, a proximal artificial graft was utilized to establish a temporary right atrial bypass loop, serving as a provisional venous drainage strategy. After the removal of the right atrial cannula, protamine was administered to neutralize heparin until ACT decreased to <150 s, and all extracorporeal circulation cannulas were removed. After 30 min of observation, no damage to coronary blood flow was observed; therefore, the temporary venous drainage maneuver was discontinued. Pericardial and mediastinal drainage tubes were placed, and the sternum was fixed with four steel wires and two sternal stabilizers after which the chest was closed in layers. There was approximately 200 mL of intraoperative blood loss. Urine output during CPB was 1,000 mL. No blood products were transfused during the procedure. The patient was transferred to the intensive care unit (ICU) postoperatively with the endotracheal tube in place. The total surgical duration was 4 h and 40 min, including 1 h and 45 min of aortic cross-clamp time and 1 h and 58 min of CPB. The results of the intraoperative arterial blood gas analysis are summarized in Table 1. Intraoperative continuous monitoring included invasive arterial and venous pressures, regional cerebral oxygen saturation (rScO_2_, 49%–57% on the left side and 52%–59% on the right side), bispectral index (BIS, 40–60), cardiac index (CI, 2.9–4.5 L/min/m^2^), and systemic vascular resistance (1,950–2,300). A pulmonary protective ventilation strategy was adopted, with a small tidal volume and positive end-expiratory positive pressure (PEEP) maintained at 6–8 cmH_2_O, as recommended for double-lung ventilation based on ideal body weight. BIS, regional cerebral oxygen saturation (rSO_2_), and PiCCO were monitored.

**Table 1:** Arterial blood gas analysis from anesthesia initiation to surgery conclusion.

Event	pH	pCO_2_ (mmHg)	pO_2_ (mmHg)	K^+^ (mmol/L)	Na^+^ (mmol/L)	Glu (mmol/L)	Lac (mmol/L)	Hct (%)	BE (mmol/L)	THbc (g/dL)	SaO_2_ (%)
Anesthesia begins	7.39	40	72	4.4	138	5.1	1.5	40	−0.7	132	94
CPB begins	7.36	47	333	5.1	132	5.3	1.0	19	1.1	59	100
CPB in progress	7.42	37	331	5.2	132	7.7	0.9	22	−0.4	68	100
Increasing temperature	7.37	39	383	5.4	133	7.9	1.5	21	−2.6	65	100
CPB completed	7.38	41	154	5.2	134	6.2	1.4	25	−0.8	78	99
Chest closure	7.36	46	169	4.5	137	5.9	1.0	29	0.3	90	99

On the first postoperative day, the patient regained spontaneous breathing and received sedation treatment. Her HR was 97 beats per minute, SpO_2_ 97%, and BP 85/51 mmHg. Circulation stabilized following infusion of 1,500 mL of fluids and transfusion of 350 mL of frozen plasma. Vital signs were maintained with HR 64–71 beats per minute, SpO_2_ 95%–98%, and BP ranging between 92 and 114/50–67 mmHg. Subsequently, sputum tuberculosis bacteria concentration culture, PCR, T-SPOT, and tuberculosis DNA testing were performed. Later that morning, the patient successfully regained consciousness and, after ventilator weaning and extubation trial, met the criteria for extubation. The tracheal tube was removed and replaced with nasal cannula oxygen therapy at a rate of 2 L/min. The patient's heart rate was 67 beats per minute, oxygen saturation was 98%, and blood pressure was 97/54 mmHg. On the second to fourth postoperative days, her vital signs remained stable and the wound drainage was normal, allowing transfer to the general ward for continued treatment. On the fifth postoperative day, follow-up cardiac ultrasound revealed no artificial vessel or perivalvular leakage. On the 10th postoperative day (16 May), the patient's artificial vessel was assessed. Chest cardiovascular CT findings confirmed post-Bentall procedure status, with no evidence of complications such as leakage or aneurysm formation. A change in cardiac size was observed compared with preoperative measurements. Pericardial effusion was reduced, and atelectasis in the region adjacent to the left lower lung showed improvement. New pleural effusion was observed in the right thoracic cavity. No other significant abnormalities were noted (Figure 2B).

**Figure 3:**
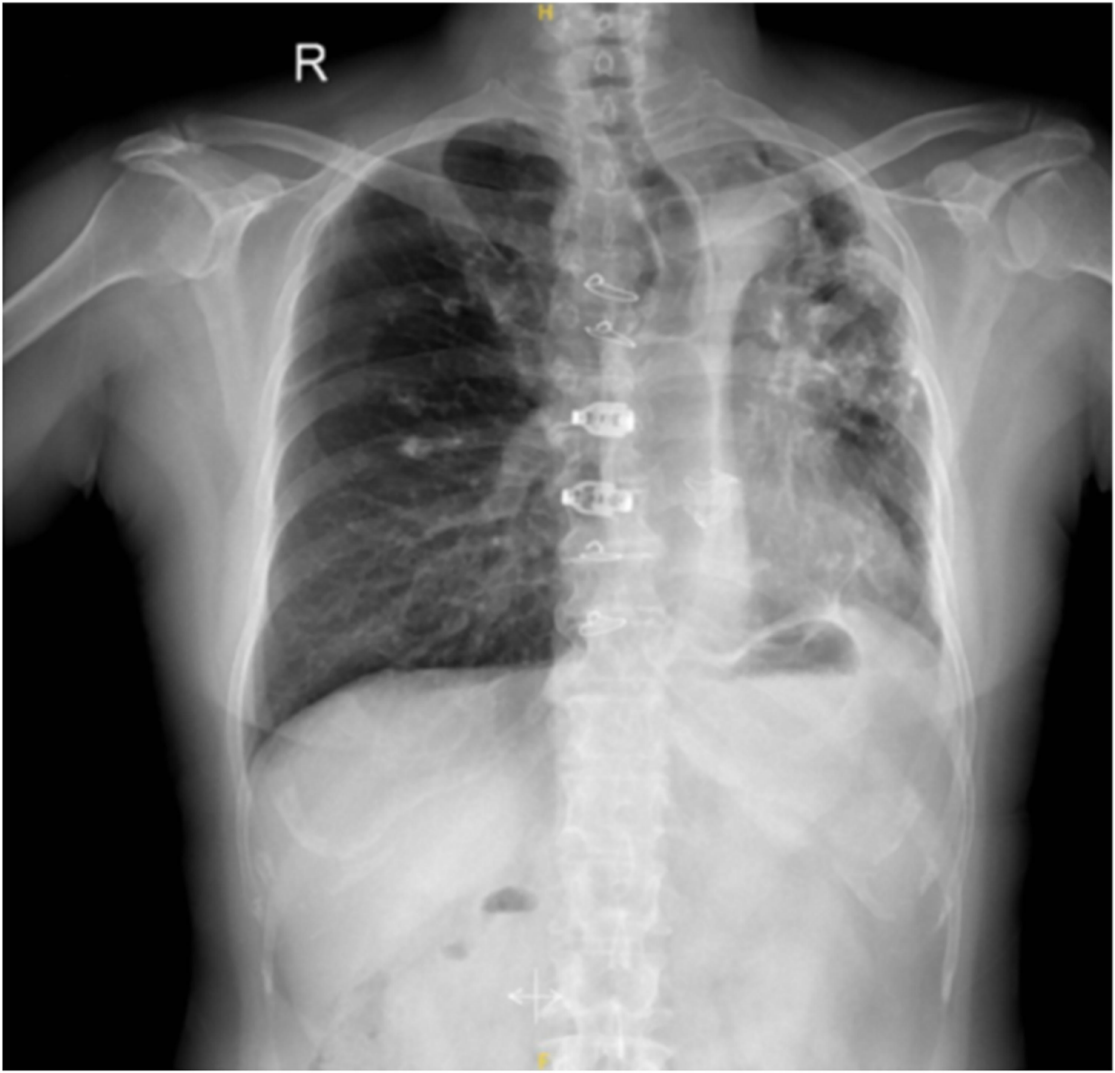
One year after operation, chest X-ray showed that the left lung reexpansion.

During this period, the patient recovered well, with no pulmonary tuberculosis dissemination observed, and the second tuberculosis test result remained negative. No postoperative pulmonary infection or other complications were observed. The patient was discharged with a favorable prognosis on the 23rd postoperative day. At the 1-month follow-up after surgery, the patient did not experience any complications related to the surgery or tuberculosis recurrence, and the chest X-ray showed no significant abnormalities (Figure 2C). During the 5-month follow-up, the patient has not reported any special surgical complications or discomfort. A follow-up chest X-ray 1 year postoperatively showed partial atelectasis compared to preoperative findings (Figure 3). The specific timeline of patient diagnosis and treatment after admission is provided in Table 2. The patient expressed gratitude to all medical staff during the postoperative year and presented a commemorative banner as a token of appreciation.

**Table 2:** Timeline of the patient's primary medical events.

Time point	Event	Key details and findings
20 years ago	Initial diagnosis and treatment of pulmonary tuberculosis	After being diagnosed with pulmonary tuberculosis, the patient discontinued anti-tuberculosis therapy after 1 year of regular treatment and has not received regular therapy since.
10 years ago	Hypertension	Long-term administration of levamlodipine 5 mg once daily for blood pressure control.
1 month before admission	The first signs of the symptoms	Unexplained cough with scant white frothy sputum, accompanied by chest tightness and dyspnea. Self-administration of “Amoxicillin + Levofloxacin” and “Salvia miltiorrhiza Tablets” resulted in symptom relief but recurrence.
1 week before admission	Symptom increased	Symptoms such as cough, chest tightness, and shortness of breath have worsened compared to before, accompanied by decreased appetite.
On admission	Visit	Due to worsening symptoms, the patient was admitted to the Department of Respiratory Medicine at Zhuzhou Hospital, Xiangya School of Medicine, Central South University.
After admission	Enhance inspections	Imaging: Enhanced chest CT revealed left lung destruction, secondary pulmonary tuberculosis in the right lung (predominantly fibrotic proliferation and calcification foci), cardiac enlargement, and aortic aneurysmal dilation (approximately 72 mm at the root). Echocardiography demonstrated left ventricular and right atrial enlargement, severe aortic regurgitation, and pericardial effusion. Laboratory findings: Elevated pro-BNP (5,267 pg/mL), negative for tuberculosis-related tests (smear, culture, PCR, T-SPOT, TB, DNA), and positive for Mycoplasma pneumoniae antibodies.
Preoperative period after admission	Multidisciplinary consultation and diagnosis	Based on the clinical presentation and examinations, the definitive diagnosis was as follows: (1) aortic root aneurysmal dilation with severe aortic regurgitation; (2) tuberculous lung destruction; and (3) mycoplasma pneumoniae infection. The assessment indicated a high risk of aneurysm rupture, with clear surgical indications.
	Preoperative preparation	The patient was initially treated for Mycoplasma pneumoniae infection. Tuberculosis-related tests were negative, with no evidence of active tuberculosis. Preoperative preparations were completed, and the Bentall procedure was planned.
On the day of the surgery	Bentall procedure	The “Bentall procedure” was performed under general anesthesia with extracorporeal circulation. During the operation, an aneurysmal dilation (approximately 72 mm in diameter) was observed at the root of the ascending aorta, along with thinning of the aortic valve leaflets and significant pericardial effusion.
Postoperative	ICU guardianship	After surgery, the patient was transferred to the ICU with stable vital signs and received sedation, ventilator support, and fluid resuscitation therapy.
Day 1 postoperatively	Successful off-line tube removal	The patient regained consciousness and successfully passed the offline test. The endotracheal tube was removed and switched to nasal cannula oxygen therapy. Circulation remained stable.
Days 2–4 postoperatively	Transfer to a general ward	The patient's condition stabilized, allowing transfer from the ICU to the general ward for continued treatment.
Day 5 postoperatively	Reexamination of cardiac ultrasound	Reexamination of cardiac ultrasound indicated that the prosthetic vessel and valve function were good, with no perivalvular leakage observed.
Day 10 postoperatively	Reexamination of chest cardiovascular CT	Postoperative changes after Bentall procedure were observed, with patent artificial vessel, reduced pericardial effusion compared to preoperative state, and cardiac size unchanged. A new right pleural effusion was noted.
Day 23 postoperatively	Rehabilitation discharge	The patient recovered well without surgical complications or tuberculosis dissemination, and was discharged successfully.
1 month postoperatively	Outpatient follow-up	The patient had no specific discomfort.
5 months postoperatively	Telephone follow-up	The patient reported no surgical complications or special discomforts.
1 year postoperatively	Long-term follow-up	The chest X-ray revealed reexpansion compared to the preoperative findings (Figure 3).

## Discussion and conclusion

According to the Global Tuberculosis Report 2023 released by the WHO, China is one of the high-burden countries for tuberculosis (4), ranking third worldwide in the number of new cases reported in 2022. According to the China CDC, the incidence of tuberculosis in China has shown a trend of decline since 1997, with an average annual decrease rate of 5.94% between 1997 and 2023. However, with the intensification of population aging, the decline in immune function among the elderly, and the combined effects of chronic diseases (such as diabetes and malignant tumors), the elderly population is more susceptible to tuberculosis (5). According to data from the China National Health Commission's Hospital Quality Monitoring System, the number of inpatients with aortic diseases in 2022 reached 128,000, accounting for 0.2% of the total number of cardiovascular disease inpatients (6). Among aortic disease patients, aortic dissection accounted for the highest proportion at 48.2%, followed by aortic aneurysm at 23.1% (7).

Both cardiovascular diseases and tuberculosis tend to affect the elderly population. With the acceleration of China's population aging, complex comorbidities such as the simultaneous occurrence of tuberculous destroyed lung and aortic aneurysm are becoming increasingly common (8). This situation poses significant challenges for diagnosis and treatment across both internal and external medicine, particularly in terms of surgical anesthesia, perioperative management, and postoperative rehabilitation, where evidence-based medical support is lacking. In this study, the patient presented with a rare clinical case of left-sided TDL combined with an ascending aortic aneurysm. To date, no studies have addressed perioperative anesthesia management and postoperative rehabilitation in such cases. There are only a few case reports on anesthesia management for tuberculous destroyed lung (9–11). The complex pathogenesis of tuberculosis is characterized by bacterial invasion, immune response, and the formation of core lesions, such as necrotic areas and cavities. Understanding these mechanisms is crucial for developing precise individualized treatment strategies for patients with tuberculous lung destruction.

TDL typically develops from pulmonary tuberculosis and is mainly caused by primary drug-resistant Mycobacterium tuberculosis infection, improper treatment, and insufficient awareness of tuberculosis prevention and control. These factors lead to ineffective control of some active pulmonary tuberculosis cases, which subsequently develop into destroyed lung (12). The clinical manifestations often include symptoms such as coughing, expectoration, hemoptysis, and difficulty breathing. The pathological changes involved in destroyed lung include fibrosis, calcification, cavity formation, and bullae in the lung tissue. These changes are irreversible and ultimately result in the loss of lung function. The disease represents a severe stage of tuberculosis, serving not only as an important source of infection but also as a major cause of death from pulmonary tuberculosis (13). Aortic aneurysms are classified into TAA (Thoracic Aortic Aneurysm) and AAA (Abdominal Aortic Aneurysm). TAA is further divided into ascending aortic aneurysms and descending aortic aneurysms. Although the incidence of ascending aortic aneurysms is lower than that of abdominal aortic aneurysms, the condition is more severe and early detection is difficult. Diagnosed often occurs only after rupture or dissection, thereby missing the optimal treatment window. Surgical difficulty and risk factors are high, and the mortality rate is extremely high (14). Although TAA and AAA have similar pathological manifestations, studies have revealed differences in pathology, molecular biology, and genetic mechanisms between the two (15, 16). The comorbid pathogenesis of coexisting tuberculous destroyed lung and ascending aortic aneurysm remains unclear and is considered rare.

Tuberculous destroyed lung is observed more commonly in female patients, with lesions often occurring on the left side. The clinical manifestations and prognosis of the disease correlate with the degree of destruction of the lung structure. In addition to clinical symptoms related to tuberculosis, patients may also exhibit symptoms such as respiratory failure, hemoptysis, recurrent lung infections, decreased exercise tolerance, and a decline in quality of life. In approximately 20%–40% of chronic progressive cases, cardiovascular complications may occur, such as pulmonary arterial hypertension and pulmonary vascular remodeling, as well as bronchial stenosis/dilation, clubbing of fingers, and concave chest on the affected side (17, 18). During the acute phase, respiratory failure may be the main clinical manifestation. If an aortic aneurysm does not invade the aortic valve ring in the early stages, the patient may have no obvious symptoms. However, if the aneurysm compresses the superior vena cava or the anonymous vein, it can cause venous distension and dilation in the neck and upper limbs. If the aneurysm grows forward and invades the sternum, the patient may experience severe pain or even protrusion of the chest wall. Once the aortic aneurysm invades the aortic valve ring, the patient often exhibits symptoms of congestive heart failure, with a diastolic murmur, widened pulse pressure, and water-hammer pulse detectable on physical examination. In the present case, the patient's tuberculous destroyed lung was fully compensated by the right lung, and the pathological changes of the aortic aneurysm were in the early to middle stages. Although the patient did not experience dyspnea, the elevated NT-proBNP level was indicative of heart failure. However, this finding is not conclusive, as it could be associated with other conditions such as cardiovascular diseases, non-cardiovascular conditions, and physiological states.

TDL resulting from contralateral lung compensation does not present any special clinical symptoms and is often diagnosed during examination for other diseases. Early X-ray manifestations include a decrease in lung volume, increased density, the mediastinum shifting toward the affected side, and the ipsilateral diaphragm being elevated. In the late stage, the affected side of the thoracic cage becomes smaller, the intercostal spaces narrow, the pleura thickens, the lung volume decreases, and the density increases. Thick-walled cavities and lung consolidation are visible, often accompanied by bullae, with the mediastinum and trachea shifting toward the affected side, and the diaphragm being elevated with adhesions. However, the clarity of the contralateral lung is often poor, requiring CT examination. CT findings of destroyed lung are basically similar to those of X-rays, but the layers and structures provide superior visualization compared with X-rays. In addition, CT can clearly display the degree of vascular and cardiac constriction. Nonetheless, it remains necessary to differentiate destroyed lung from congenital polycystic lung and bronchial foreign bodies (widespread fibrosis type) (11, 18). Asymptomatic ascending aortic aneurysms are generally difficult to detect and are often found and diagnosed during physical examinations or other related examinations for other diseases. Plain X-ray films can show calcified aneurysm walls, and further CT angiography or MRI can clarify the location, extent, and size of the aneurysm. Ultrasound can measure the size, pulsation, and murmur of the aneurysm. Physical examination, such as the reactive hyperemia test, can also be used to observe whether circulation of the affected limb is established. It is important to protect the heart, brain, spinal cord, and internal organs from ischemic and hypoxic damage, and prevent acute enlargement and failure of the left ventricle due to blocked blood flow. Once a clear diagnosis is established, surgical intervention should be performed as soon as possible, particularly for patients with destroyed lungs and aortic insufficiency, where Bentall surgery should be performed promptly. In the present case, the patient was hospitalized for coughing, expectoration, chest tightness, and shortness of were observed, and examination revealed left lung destruction with an ascending aortic root aneurysm. Following multidisciplinary consultation, the patient successfully underwent Bentall surgery under elective extracorporeal circulation, and she then demonstrated good postoperative recovery through rapid rehabilitation.

As China's aging population intensifies, approximately 42.4%–81.3% of elderly individuals face the distress of multiple coexisting diseases. Moreover, patients with multiple diseases—such as destroyed lung and ascending aortic aneurysm—present significant challenges for perioperative anesthesia management during cardiovascular surgery. Although many perioperative risk assessment scales include comorbidities, they merely list the number of chronic diseases without accurately predicting perioperative risk and prognosis (19). The Charlson Comorbidity Index (CCI), widely used to predict mortality risk, assigns different weights to specific diseases. While specific diseases are addressed, there are shortcomings in disease coverage, weight distribution, and the interaction between diseases (20). For patients with thoracoabdominal aortic dissection (TDL) and ascending aortic aneurysms, multidisciplinary discussions were initiated. After assessment using the CCI and the cardiac surgery risk assessment EuroScore II, Bentall surgery and aortic valve replacement were scheduled, considering the high success rates and the expertise of the surgical team. Given the patient's destroyed lung, inhaled anesthesia was not used. Smeltz et al. have suggested that total intravenous anesthesia is superior to combined intravenous and inhaled anesthesia in cardiac surgery (21). Studies have shown that the lung-protective ventilation strategy (LPVS), which involves the application of low tidal volume (6–8 mL/kg) and low end-expiratory positive pressure (PEEP <5 cmH₂O) combined with lung re-expansion techniques during cardiac surgery, is beneficial for reducing postoperative pulmonary complications (22). Therefore, LPVS can reduce the incidence of postoperative pulmonary complications, but higher PEEP may lead to pulmonary edema (23). A systematic review by Guo et al. (2018) found that higher levels of PEEP (5–8 cmH_2_O) combined with small tidal volumes can effectively reduce lung injury caused by mechanical ventilation and prevent atelectasis, particularly in acute respiratory distress syndrome (ARDS) patients. In addition, TDL patients often have pulmonary hypertension, so it is necessary to avoid intraoperative factors that cause acute pulmonary vasoconstriction, such as hypoxia, hypercapnia, and acidosis (24).

After adopting the ERAS strategy for the patient in the present case, she was successfully extubated on the second postoperative day and could walk with the aid of a device. A CT scan on the sixth postoperative day showed partial reinflation of the collapsed left lung tissue (Figure 2B), possibly attributable to postoperative lung inflation or the use of PEEP. Further examination and evaluation were needed to determine if lung function had recovered. Studies have demonstrated that mechanical ventilation is an effective treatment for refractory respiratory failure in TDL patients, significantly improving their condition (25). Intraoperative blood gas analysis revealed an oxygen partial pressure of 154 mmHg (oxygen concentration 60%), and postoperative ICU monitoring showed an oxygen partial pressure of 185 mmHg (oxygen concentration 40%), indicating that general anesthesia mechanical ventilation acts as a form of treatment. The patient recovered well and was discharged on the seventh postoperative day. At the 6-month follow-up, the patient had returned to preoperative levels, with no related complications.

For patients with tuberculous lung destruction complicated by aortic root aneurysm undergoing Bentall surgery under CPB, anesthesiologists face significant challenges in circulatory and respiratory management. Comprehensive preoperative evaluation—encompassing infection control, nutritional support, cardiopulmonary function assessment, and medication adjustment—is essential for patients with tuberculosis to ensure safe surgical outcomes. Such cases necessitate a comprehensive preoperative evaluation, including fiberoptic bronchoscopy, pulmonary function testing, cardiac ultrasound, and cardiovascular angiography. Detailed assessment must be conducted to determine whether the aneurysmal dilation involves the valves or causes valvular pathology, and whether the Bentall procedure should involve valve-preserving root replacement or simultaneous replacement of the valve and ascending aorta with artificial vessels and valves. Intraoperative and postoperative resuscitation should actively incorporate lung-protective ventilation strategies. At present, there is a lack of systematic clinical research on perioperative anesthesia management for patients with TDL complicated by cardiovascular disease. The potential correlation between the pathogenic mechanisms of these two conditions remains unclear. Further basic research and large-scale clinical studies are needed to elucidate the specific mechanisms and perioperative management strategies. Evidence from experiences with patients suffering from tuberculous destroyed lung and elderly patients demonstrates that tailored perioperative care protocols can significantly improve clinical outcomes.

## Data Availability

The original contributions presented in the study are included in the article/Supplementary Material, further inquiries can be directed to the corresponding author.
